# A case of a common bile duct stone that formed around a fish bone as a nidus after distal gastrectomy with Roux-en-Y reconstruction

**DOI:** 10.1186/s40792-021-01142-6

**Published:** 2021-02-25

**Authors:** Ken Hirata, Daichi Kawamura, Masahiko Orita

**Affiliations:** Department of Surgery, Hikari Municipal Hikari General Hospital, Hikarigaoka 6-1, Hikari, Yamaguchi 743-8561 Japan

**Keywords:** Foreign body, Fish bone, Common bile duct stone, Gastrectomy, Roux-en-Y reconstruction

## Abstract

**Background:**

The presence of a foreign body in the common bile duct (CBD) is a rare phenomenon. Thus, the route and mechanism of its migration remain difficult to fully clarify, especially for cases that occur after gastrectomy with Roux-en-Y reconstruction. Herein, we present a case of a CBD stone that formed around a fish bone as a nidus subsequent to distal gastrectomy with Roux-en-Y reconstruction.

**Case presentation:**

A 70-year-old man was admitted to our hospital due to repeated episodes of epigastralgia. He had undergone distal gastrectomy with Roux-en-Y reconstruction for gastric cancer approximately 10 years prior. Blood tests revealed obstructive jaundice, hepatobiliary dysfunction, and inflammation. Multi-plane reconstructed computed tomography (CT) revealed a CBD stone with a needle-shaped calcification density at the center, oriented along the length of the CBD. Surgery was performed using an upper median laparotomy approach. Lithotomy with choledochotomy was performed to remove one fragile bilirubin stone that had formed around a 3-cm, needle-shaped fish bone. A choledochoduodenal fistula was not detected intraoperatively. A review of the imaging of a prior examination revealed that the formation of the CBD stone around the fish bone was observable on a follow-up CT performed approximately 2 years prior. However, no clinical symptoms associated with the migration of the fish bone to the CBD were reported and the fish bone was not detected at that time.

**Conclusion:**

In this case, transpapillary migration of the fish bone could only be speculated in the absence of an observable fistula, choledochostomy, or any clinical symptoms. Our case is clinically relevant as cholangitis developed after CBD stone formation around the fish bone that acted as a nidus.

## Background

The presence of foreign bodies in the common bile duct (CBD) is a rare phenomenon. Although reports of such cases are gradually increasing, the route and mechanism of migration of such foreign bodies into the CBD remain to be clarified. Additionally, there are no reports about the length of time required to form a stone after foreign body migration into the CBD. As such, there is a need for more case reports to be accumulated and discussed. Herein, we present a case of a CBD stone that formed around a fish bone as a nidus after distal gastrectomy with Roux-en-Y reconstruction.

## Case presentation

**Fig. 1:**
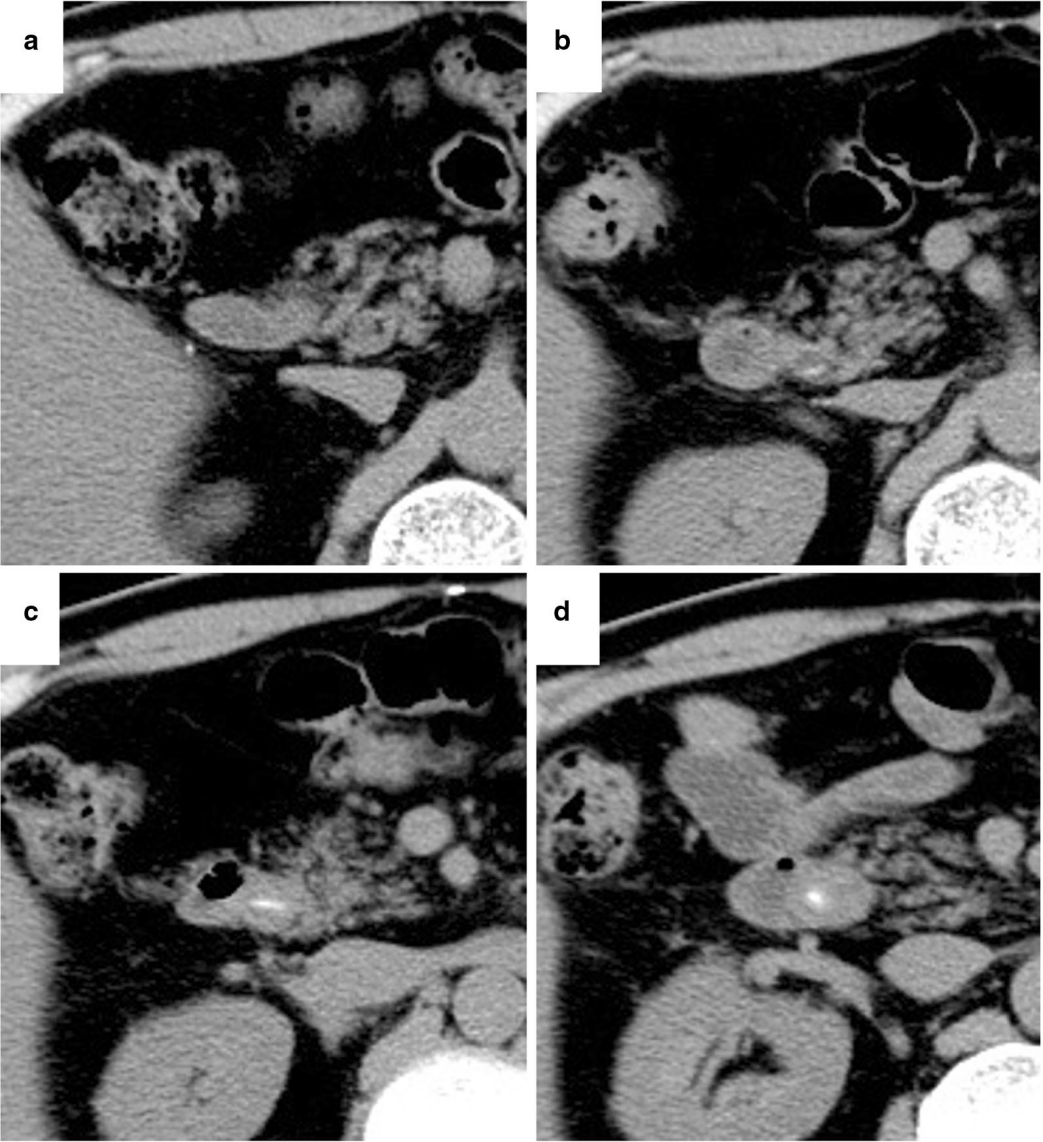
The time series of the surveillance computed tomography (CT) after gastrectomy. In 2012, 7 years after the patient underwent distal gastrectomy with Roux-en-Y reconstruction for gastric cancer, the common bile duct (CBD) stone was not detected (**a**). In 2015, at the past CT scan review, a spotted high-density area was recognized (**b**). The stone formation was also recognized in 2016, although the patient had no symptoms (**c**). CBD dilatation with stone formation causing obstructive jaundice was detected in 2017 (**d**)

**Fig. 2:**
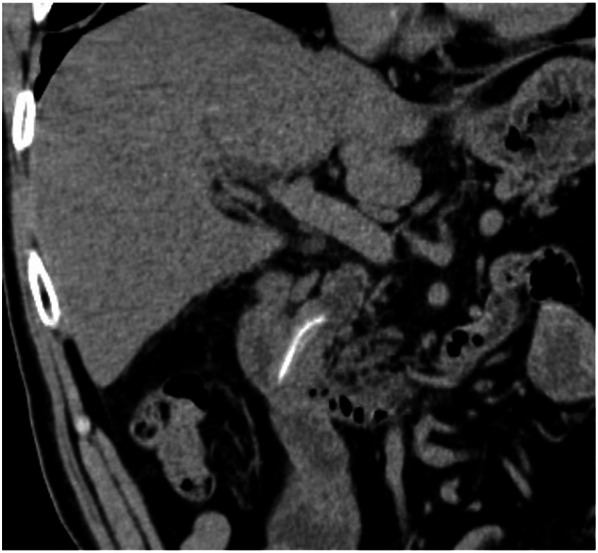
The coronal plane computed tomography (CT) image showing a common bile duct (CBD) stone along the length of the CBD, with a needle-shaped calcification density at the center

**Fig. 3:**
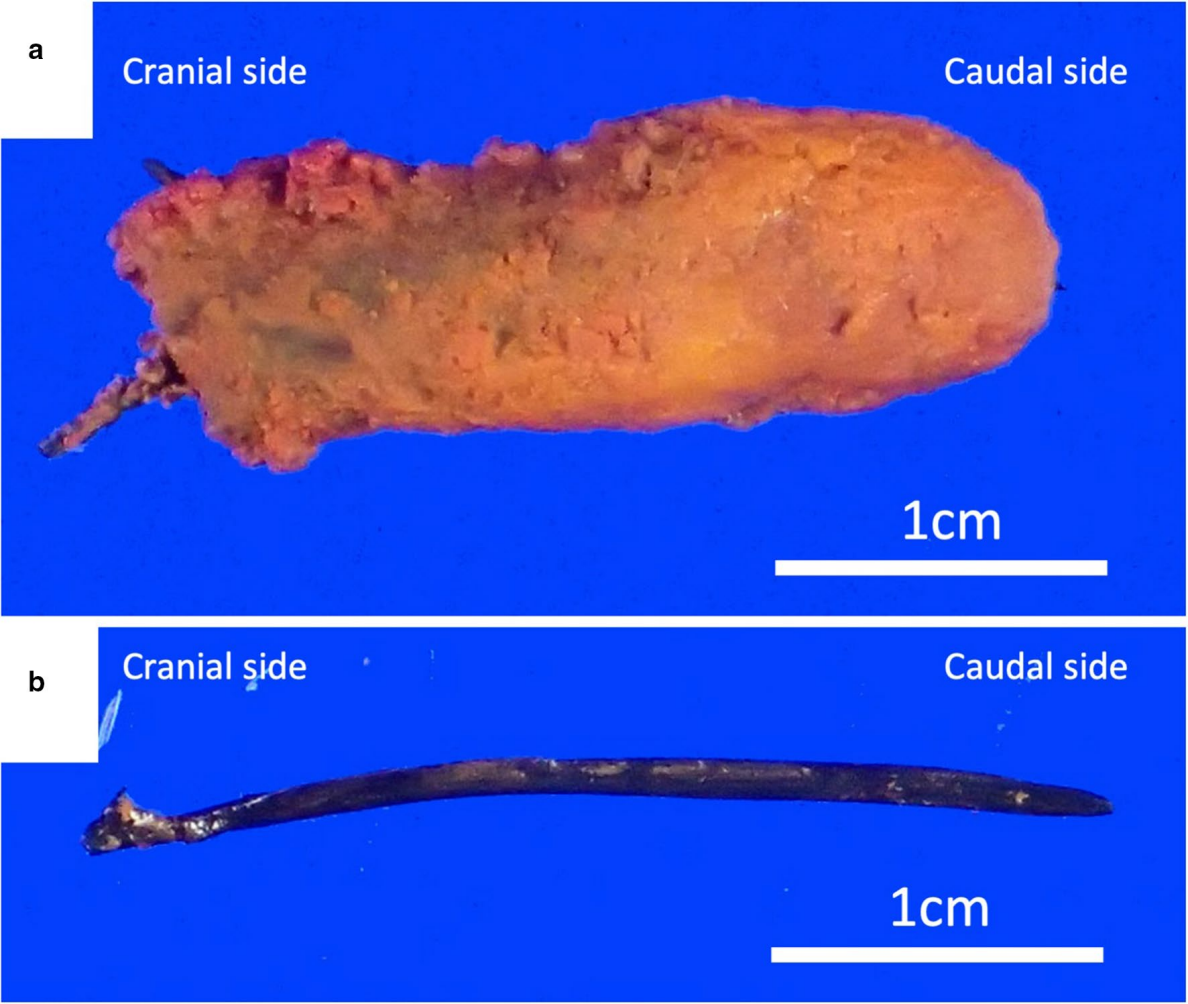
The extracted common bile duct (CBD) stone. Insoluble calcium salts aggregated and formed stones around the fish bone that acted as a nidus (**a**). The fish bone was approximately 3 cm in length (**b**)

**Fig. 4:**
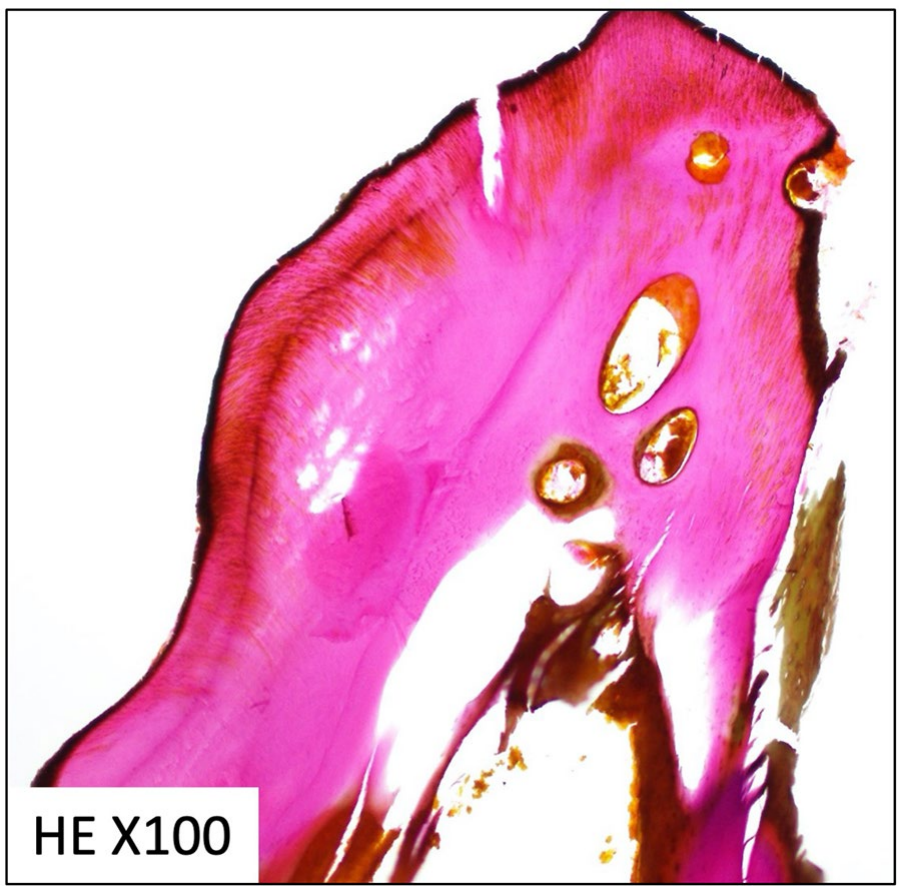
Histological hematoxylin–eosin (HE) staining of the needle-shaped foreign body. The bone matrix that was constructed with collagen fibers was dyed red with hematoxylin

A 70-year-old man was admitted to our hospital for obstructive jaundice. He had undergone distal gastrectomy with Roux-en-Y reconstruction for gastric cancer concomitant with cholecystectomy for cholecystolithiasis approximately 10 years prior at a different hospital. The pathological staging of his gastric cancer was SS, N0, M0, stage IB. Gastric cancer recurrence was not detected during postoperative surveillance by computed tomography (CT) imaging or blood examination (Fig. 1a). In November 2016, further examination for repeated episodes of epigastralgia revealed the presence of obstructive jaundice. The patient’s serum total and direct bilirubin levels were 4.2 and 2.4 mg/dL, respectively. Moreover, hepatobiliary dysfunction was detected and the patient’s serum alkaline phosphatase, aspartate aminotransferase, alanine aminotransferase, and gamma-glutamyl transferase levels were 474 IU/L, 264 IU/L, 496 IU/L, and 676 IU/L, respectively. C-reactive protein was elevated at 11.2 mg/dL. CT examination revealed CBD dilatation with a high-density area (Fig. 1d). On a series of surveillance CT images, a spotted high-density area in the CBD was confirmed in a retrospective analysis following the onset of cholangitis (Fig. 1b, c). Multi-plane reconstructed (MPR) CT revealed a CBD stone along the length of the CBD that contained a needle-shaped calcification density at its center (Fig. 2). The patient was diagnosed as acute cholangitis concomitant with CBD stone. Gastroenterologists at our center and from a nearby hospital deemed that it would be difficult and tentative to perform endoscopic therapy to remove the stone through the Roux-en-Y reconstructed intestine. Therefore, the surgical procedure was adopted as quick and reliable treatment option and was performed using the upper median laparotomy approach. A severe adhesion, resulting from prior surgeries (namely distal gastrectomy and cholecystectomy), was found between the liver and the hepatoduodenal ligament. Even though the CBD was carefully exposed to adhesiolysis, a choledocoduodenal fistula was not detected. Lithotomy with choledochotomy was performed to remove one fragile bilirubin stone that had formed around a 3-cm, needle-shaped fish bone (Fig. 3). The choledochotomy was closed by interrupted sutures using a polyglactin 910 suture. CBD drainage was not performed. The needle-shaped foreign body was diagnosed as a fish bone by histological hematoxylin and eosin staining and infrared absorption spectrophotometry (Fig. 4). The patient’s postoperative course was uneventful and free of adverse events. The patient was discharged on postoperative day 21. No recurrence of a CBD foreign body was identified over the 4 years since the surgery.

## Discussion

In this case, we confirm the possibility of a CBD stone forming around a fish bone as a nidus. Moreover, in the absence of an observable fistula, the fish bone was assumed to have migrated into the CBD, although the route and mechanism of this migration remain to be clarified.

We summarize previously published cases on CBD foreign bodies in Japan. We identified 54 reports (of 63 cases) that identified the following causes: iatrogenic (42/63, 66.7%), oral ingestion (17/63, 27.0%), extracorporeal penetration (1/63, 1.6%), and parasites (3/63, 4.8%). In the world, foreign bodies introduced by ingestion have included toothpicks [1, 2], needles [3], fish bones [4, 5], chicken bones [6], and plant fibers [7, 8]. Due to the high fish consumption in Japan, a fish bone was the most identified foreign body in the CBD in 16 cases, including our case [4, 9–19], and plant fiber in one cases [20] (Table 1).

Choledochoduodenal fistulae and post-biliary jejunostomies have been reported as routes of foreign body migration into the CBD [1, 5]. Other possibilities include direct access by needle-shaped objects, such as a fish bone [1, 4, 7], transhepatic migration [21], and transpapillary regurgitation [2, 3, 8, 10, 22, 23]. There is also the possibility of excretion of foreign bodies from the liver into the bile duct, as shown in a case of a shrapnel splinter in the right thoracic cavity that migrated into the CBD through the diaphragm and liver [24]. Foreign bodies could also migrate into the CBD through the papilla, as shown by cases in which soft material impaction, such as plant stems or fibers and a chicken bone, in the duodenal papilla was the contributing source of the CBD foreign body [6, 8, 20].

In our case, after distal gastrectomy and Roux-en-Y reconstruction, severe adhesions were found between the residual stomach and liver, as well as around the duodenal stump and hepatoduodenal ligament, although there was no fistula or jejunostomy. Additionally, no previous duodenal papillae treatment had been performed. Thus, besides the transpapillary route, transient gastrohepatic, and duodenal biliary fistulae were considered as possible causes but with no conclusive evidence. During the clinical course, since the fish bone migrated into the bile duct, there were no specific abnormalities, such as pain or inflammation associated with perforation. As such, we considered transpapillary foreign body migration as the most likely route of migration, as previously described [25]. In six cases (37.5%) reported in Japan, migration through the duodenal papilla was considered [10, 15–19]. The introduction of an ingested foreign body into the bile duct via reflux should also be considered, regardless of duodenal papilla treatment [7, 10]. In cases of post-gastrectomy, abnormal motility of the sphincter of Oddi has been observed by severing the intrinsic neural connection from the stomach at the proximal duodenum. As evidenced by the paradoxical response to cholecystokinin, post-gastrectomy sequelae may stimulate the contraction of the sphincter of Oddi. But it has not been clarified how a motor disorder of the sphincter of Oddi is related to regurgitation [26]. A study investigating reflux of oral contrast agents into the bile ducts during magnetic resonance cholangiopancreatography identified prior intervention to the duodenal papilla, parapapillary diverticulum, and pneumobilia as risk factors for reflux, although gastrectomy was not associated with the development of reflux [27].

Once the physiological analysis is completed, it is followed by the anatomical analysis. As several of the ingested foreign bodies reported to date have a thin needle-like shape and are approximately a few centimeters long, we hypothesize that they may be easily caught in the duodenal folds by peristalsis when passing through the curvature of the duodenum. Given that the duodenum is fixed to the retroperitoneum, the shape of the duodenum is almost flat in the direction of the papillary side of the small curvature, opposite to the greater curvature. Therefore, a long and thin object, such as a fish bone, that moves along a diagonal line in the major axis of the duodenal may unexpectedly be caught in the duodenal papilla.

Several hypotheses have been put forth as to why a fish bone is not expulsed from the CBD. The tip of the fish bone may have become lodged in the wall of the bile duct, subsequently being pushed into the bile duct by peristalsis, even if exposed to the duodenal papilla. Bile stasis might also be an associated factor. However, clear research evidence regarding these routes is needed. There is also the possibility of reflux into the afferent loop due to distal intestine stenosis or obstruction; however, such symptoms were not observed in our patient. Migration of the foreign body from the afferent loop to the duodenum, insertion into the duodenal papilla, and its persistent location in the bile duct appear to be coincident.

In our case, a retrospective review of prior images identified that the process of CBD stone formation around the fish bone took approximately 2 years. The fish bone initially stagnated in the CBD, without stone formation, for the first year. Subsequently, a CBD stone gradually grew around the fish bone and finally became incarcerated for approximately 2 years, causing acute cholangitis. Prior studies have shown the potential for food-based foreign bodies in the CBD to act as a nidus for stone formation, making excretion difficult [1, 9]. In our case, the stone that formed around the fish bone was formed of ocher bilirubinate. Bilirubin becomes deconjugated by the bacterial infection associated with cholestasis, resulting in the aggregation of poorly soluble calcium salts that led to stone formation around the fish bone [23]. Stone formation associated with a bacterial infection in the CBD is suggestive of reflux from the duodenal papillae.

In various reports, CT imaging has been useful to identify aspirated fish bones [9, 12, 28]. In our case, the fish bone in the CBD was visible on coronal plane MPR CT images performed at the onset of the cholangitis. On images of the axial plane CT, only a dot-like high-density area was observable, even though it could not be confirmed as an abnormality as the patient did not develop any symptoms. In particular, it was difficult to detect the very small spotted high-density area on the axial plain, which was performed before the cholangitis as a periodical examination. Today, owing to improvements in CT processing speed and resolution, CT reconstructed imaging could be routinely performed in cases with biliary system abnormalities.

Although endoscopic treatment is currently a well-established procedure, the patient would more likely be treated initially by single- or double-balloon endoscopy [9, 12]. However, this would have been technically difficult to perform in our case due to the altered anatomy following the Roux-en-Y reconstruction. The success rate of reaching the duodenal papilla would still be low especially in the presence of a residual stomach, and shortly after the introduction of the new procedure at the institution. Therefore, we proceeded with open surgery as the more reliable treatment option during which severe adhesions were carefully dissected, although the procedure itself was the same as for normal bile duct stones and, therefore, there were no safety issues.

In our case, as a clinical experience, there was a progression from the invasion of the fish bone into the CBD to stone formation and the onset of cholangitis. However, though we speculate on a transpapillary pathway of migration of the fish bone into the CBD, there was no confirmatory evidence. There is a need to accumulate cases and experiences to improve our understanding of the process of foreign body invasion of the CBD.

## Conclusions

We describe a rare case of a CBD stone that formed around a fish bone as a nidus after distal gastrectomy with Roux-en-Y reconstruction. In this case, transpapillary migration of the fish bone was considered as the route of entry, in the absence of a fistula, choledochostomy, or any clinical symptoms. Our case is valuable because the process of cholangitis development was retrospectively observed after the CBD stone had formed.

**Table 1:** The 16 cases of fish bones in the common bile duct reported in Japan

No.	Author	Year	Age	Sex	Chief complaints	Previous disease and interventional treatment or surgery	Interval time of migration (years)	length of fish bone (mm)	Therapeutic procedures for CBD stone	Possible pathway of migration to CBD
1	Morimoto	2018	73	F	Rt. hypochondralgia Back pain	None	–	35	EST	Through the papilla by chance
2	Sakakida	2018	78	F	Fever Epigastric pain	Duodenal carcinoma Pancreaticoduodenectomy	9	20	Single-balloon endoscopic lithotomy	Choledochojejunostomy
3	Koga	2018	71	M	Fever	Cholangiocarcinoma Pancreaticoduodenectomy	1	Not specified	Double-balloon endoscopic lithotomy	Choledochojejunostomy
4	Bamba	2017	71	M	Fever Liver dysfunction	Intraductal papillary mucinous neoplasm Pancreaticoduodenectomy	2	25	Single-balloon endoscopic lithotomy	Choledochojejunostomy
5	Kuga	2016	63	M	Fever Epigastric pain	Perforation of juxtapapillary duodenal diverticulum Pylorus-preserving pancreaticoduodenectomy	5	19	Laparotomy	Choledochojejunostomy
6	Araya	2016	79	F	Epigastric pain Jaundice	None	–	15	EST	Not specified
7	Hori	2016	70	M	Consciousness disturbance Fever	1. Distal gastrectomy, Billroth II reconstruction 2. CBD stone, choledochoduodenostomy	40 5	25	Laparotomy	Choledocoduodenostomy
8	Yoshida	2010	73	M	Rt. upper abdominal pain Fever	CBD stone, EST	3	40	EST	Through the papilla (post-EST)
9	Yoshida	2005	77	M	Rt. hypochondralgia	None	-	38	Endoscopic papillary balloon dilatation	Through the papilla by chance
10	Kaji	2004	83	M	Jaundice	None	-	30	Laparotomy Choledochotomy	Temporary choledochoduodenal fistula
11	Watanabe	1999	74	F	Fever (ampulla carcinoma of Vater)	None	-	30	Laparotomy Pancreaticoduodenectomy	Through the papilla by chance
12	Sakai	1997	69	M	Rt. hypochondralgia	CBD stone, EST	1	32 and 44	Endoscopic lithotomy	Through the papilla (post-EST)
13	Kakiuchi	1989	63	M	Epigastric pain	Gastric cancer, gastrectomy	16	25	Laparotomy Choledochotomy	Through the papilla by chance
14	Kato	1985	84	M	Epigastric pain Vomiting	Gastric ulcer, gastrectomy	30	40	Laparotomy Choledochotomy	Not mentioned
15	Sato	1980	60	F	Epigastric pain Jaundice	Gastric ulcer, gastrectomy, Billroth II reconstruction	10	50	Laparotomy Choledochotomy	Not mentioned
16	Our case	2017	70	M	Epigastric pain	Gastric cancer Distal gastrectomy, Roux-en Y reconstruction	2	32	Laparotomy Choledochotomy	Through the papilla by chance (speculated)

The interval time of migration is defined as the time interval for the migration after a previous interventional treatment or surgery and is shown as the number of years

*F* female, *M* male, *Rt* right, *CBD* common bile duct, *EST* endoscopic sphincterotomy

## Data Availability

All data presented in this paper are available upon request.

## Abbreviations

CBD: Common bile duct
FB: Foreign body
CT: Computed tomography
MPR: Multi-plane reconstruction
HE: Hematoxylin–eosin
